# Comparative transcriptomic and proteomic analysis of yellow shell and black shell pearl oysters, *Pinctada fucata martensii*

**DOI:** 10.1186/s12864-019-5807-x

**Published:** 2019-06-08

**Authors:** Meng Xu, Jing Huang, Yu Shi, Hua Zhang, Maoxian He

**Affiliations:** 1 CAS Key Laboratory of Tropical Marine Bio-resources and Ecology, Guangdong Provincial Key Laboratory of Applied Marine Biology, South China Sea Institute of Oceanology, Chinese Academy of Sciences, Guangzhou, 510301 China; 2 University of Chinese Academy of Sciences, Beijing, 100049 China

**Keywords:** Transcriptomics, Proteomics, Calcium metabolism, Carotenoids, Yellow shell colour pigmentation, *Pinctada fucata martensii*

## Abstract

**Background:**

The pearl oyster *Pinctada fucata martensii* (*Pfu.*), widely cultured in the South China Sea, is a precious source of sea pearls and calcifying materials. A yellow shell variety of *Pfu.* was obtained after years of artificial breeding. To identify differentially expressed genes between yellow shell and normal black shell pearl oysters, we performed transcriptomic sequencing and proteomic analyses using mantle edge tissues.

**Results:**

A total of 56,969 unigenes were obtained from transcriptomic, of which 21,610 were annotated, including 385 annotated significant up-regulated genes and 227 significant down-regulated genes in yellow shell oysters (| log_2_ (fold change) | ≥2 and false discovery rate < 0.001). Tyrosine metabolism, calcium signalling pathway, phototransduction, melanogenesis pathways and rhodopsin related Gene Ontology (GO) terms were enriched with significant differentially expressed genes (DEGs) in transcriptomic. Proteomic sequencing identified 1769 proteins, of which 51 were significantly differentially expressed in yellow shell oysters. Calmodulin, N66 matrix protein, nacre protein and Kazal-type serine protease inhibitor were up-regulated in yellow shell oysters at both mRNA and protein levels, while glycine-rich protein *shematrin-2*, mantle gene 4, and sulphide: quinone oxidoreductase were down-regulated at two omics levels. Particularly, calmodulin, nacre protein N16.3, mantle gene 4, sulphide: quinone oxidoreductase, tyrosinase-like protein 3, cytochrome P450 3A were confirmed by quantitative real-time PCR. Yellow shell oysters possessed higher total carotenoid content (TCC) compared than black shell oyster based on spectrophotography.

**Conclusions:**

The yellow phenotype of pearl oysters, characterised by higher total carotenoids content, may reflect differences in retinal and rhodopsin metabolism, melanogenesis, calcium signalling pathway and biomineralisation. These results provide insights for exploring the relationships between calcium regulation, biomineralisation and yellow shell colour pigmentation.

**Electronic supplementary material:**

The online version of this article (10.1186/s12864-019-5807-x) contains supplementary material, which is available to authorized users.

## Background

Shell colour polymorphism, a common qualitative characteristic of shellfish, has been investigated using various chemical and molecular biological methods, but many issues remain unsettled, including the relationship between shell colour and material properties [1]. The shell of the pearl oyster *Pinctada fucata martensii* (*Pfu.*) consists of a periostracum layer, a prismatic layer containing calcite, and a nacre layer containing aragonite, from outer to inner layers [2, 3]. Calcium carbonate (CaCO_3_) is a key mineral phase in this highly sophisticated and complex structure, and its synthesis depends on the absorption of calcium. Recent reports revealed that the origin of shell colour in the related species *Pinctada margaritifera* is associated with biomineralisation of the calcitic layer [4]. Two cDNA suppression subtractive libraries constructed for red-shelled and non-red-shelled *Pfu.* revealed that genes encoding shematrin, mantle protein, and nacrein are related to shell colour [5]. Gong [6] found that an increased concentration of calcium ions can enhance nacrein secretion. Pigments in higher molluscs such as bivalves are thought to be tightly attached to conchiolins (organic matrix proteins) in the shell, similar to gastropods and pulmonarias [7], while the periostracum layer is composed of conchiolins and calcium salts. Calmodulin and calmodulin-like protein, two important proteins in calcium transport and secretion processes, regulate calcite growth and aragonite nucleation in bivalves [8–11]. Sun et al. [8] found that calmodulin-related protein, adenylate cyclase, and tyrosinase family members are involved in both biomineralisation and melanin biosynthesis in scallop *Patinopecten yessoensis*. Another study showed that the notch signalling pathway plays a vital role in shell pigmentation in the clam *Meretrix meretrix*, while calcium signalling may activate this pathway and influence shell colour [12]. Sequential layer-by-layer mineralisation is directed by cells of the mantle edge in *Pfu*., and pigments, glycoproteins and polysaccharides in the periostracum layer are secreted by the mantle or foot tissue in molluscs [13, 14]. Thus, there may be a close relationship between biomineralisation and pigment deposition, with some genes acting as a bridge between these two biological phenomena.

Studies have shown that melanin [7], porphyrins [7, 15], bile pigments [16] and carotenoids [17] are the main pigments in mollusc shells [1]. Melanin is a common pigment found in bacteria, plants, fungi and higher animals that performs diverse function related to growth promotion, immune defences, stress resistance, and antioxidation [8, 18]. The 3,4-dihydroxyphenylalanine (DOPA) intermediate in melanogenesis is important for periostracum layer sclerotisation, and affects quinone tanning of the periostracum in the bivalve *Perna viridis* [1, 19]. Quinone tanning is believed to be an essential prerequisite for orderly deposition of calcium carbonate crystals [20, 21]. Ogimura et al. [22] suggested the black spots on the shells of pearl oysters may be related to melanin, and the melanin pathway may perform a defensive role against pathogen infection and inflammatory reaction. Carotenoids perform similar biological functions to melanin, acting as antioxidants and supporting the immune system [23, 24]. Li et al. [25] identified the novel new carotenoid pectenolone in muscle of the Yesso scallop *Patinopecten yessoensis*. Furthermore, a high total carotenoid content (TCC) can enhance tolerance to high temperatures in *Pfu.* [26], and total antioxidant capacity (TAC) in the noble scallop *Chlamys nobilis* [27, 28].

**Fig. 1:**
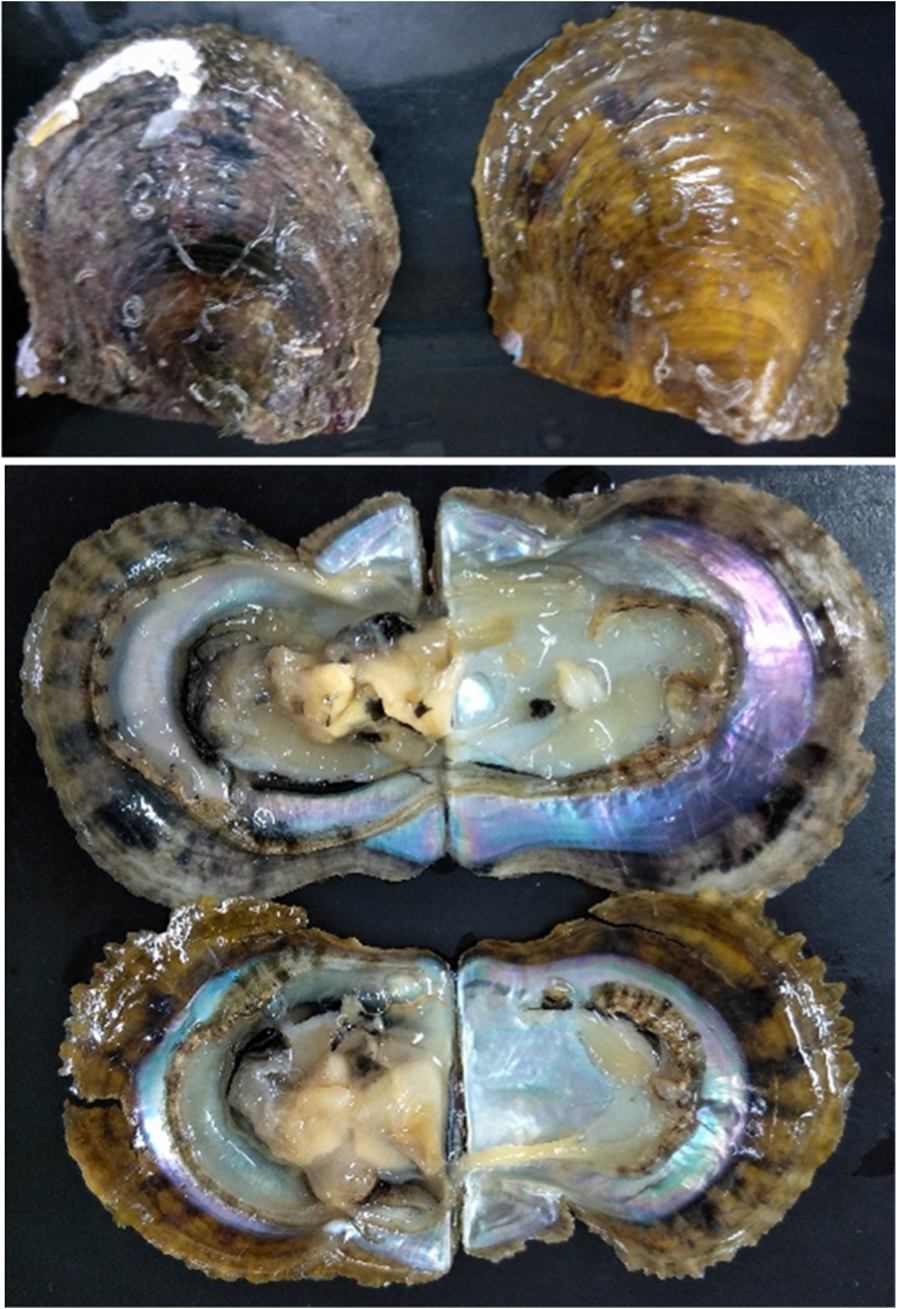
Photographs of black shell and yellow shell pearl oyster (*Pinctada fucata martensii*).The yellow phenotype has yellow pigmentation in both the periostracum layer and prismatic layer.

Yellow shell colour lines of *Pfu.* showed significant differences in shell and weight index [29], and can affects growth traits [30] and pearl quality [31]. However, the mechanism of yellow shell formation is not clear. In the present study, comparative transcriptomic and proteomic analysis was performed on mantle edge tissue from yellow shell and black shell *Pfu.* Differentially expressed genes (DEGs) in the two shell colour phenotypes (Fig. 1) were identified and characterised by bioinformatics and functional annotation. The findings lay a foundation for investigating the mechanism of yellow shell pigmentation.

## Results

### TCC values for different tissues from yellow- and black-shelled pearl oysters

**Fig. 2:**
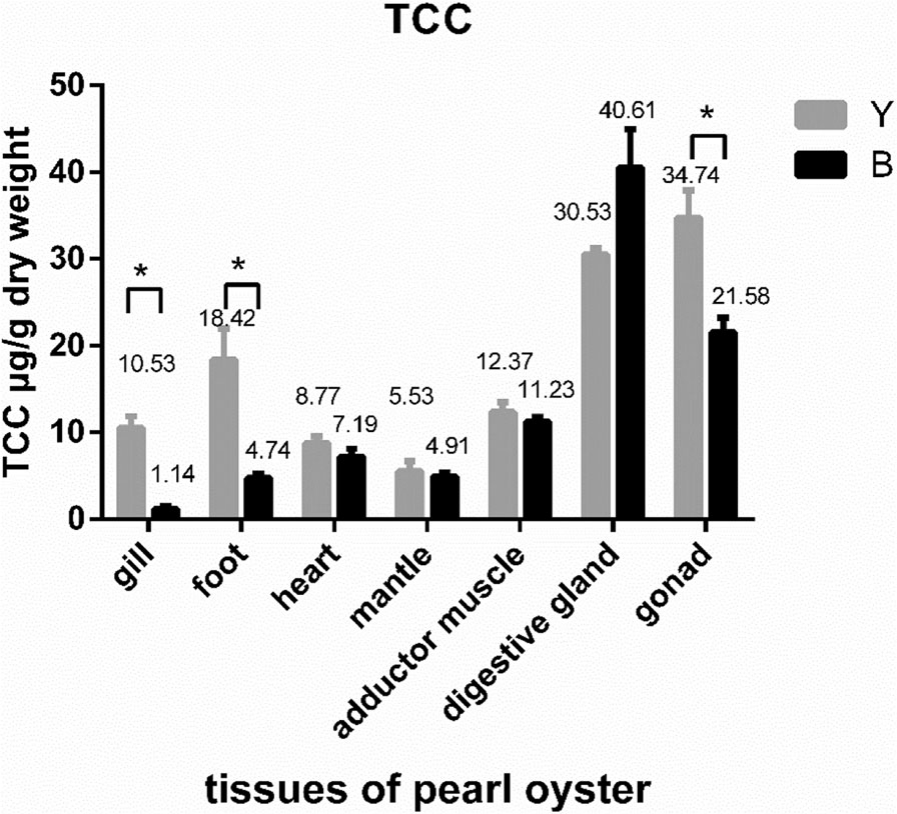
TCC in different tissues of yellow shell and black shell oyster groups.TCC, total carotenoid content; Y, yellow shell color pearl oysters; B, black shell color pearl oysters.

TCC values ranged from 5.53 to 34.74 μg/g dry weight across seven different tissues from yellow oysters (Y), with the highest value in gonad tissue. TCC values ranged from 1.14 to 40.61 μg/g dry weight across seven different tissues from black oysters (B), with the highest value in digestive gland tissue (Fig. 2). TCC values in gill, foot, heart, adductor muscle, mantle, digestive gland and gonad from group B samples were 1.14, 4.74, 7.19, 4.91, 11.23, 40.61 and 21.58 μg/g dry weight, respectively, compared with 10.53, 18.42, 8.77, 5.53, 12.37, 30.53 and 34.74 μg/g dry weight for group Y tissues.

The average TCC value across the seven different tissues was 17.27 μg/g dry weight for group Y samples, somewhat higher than that of group B samples (13.06 μg/g). TCC values of gill, foot and gonad tissues from group Y were significantly higher than those from B (*p* < 0.05), and TCC values of heart, mantle and adductor muscle were also higher for group Y samples.

### Transcriptome analysis and pathways related to yellow shell colour

In total, 98.05% of raw reads were clean reads in T01 (Black, B) and 98.53% of raw reads were clean reads in T02 (Yellow, Y). Two cDNA libraries were constructed with mantle tissues from Y and B pearl oysters, yielding 18.78 Gb of clean data (NCBI accession number: SRR8357272 for B, SRR8357273 for Y) with Q_30_ ≥ 89.82% for Y and Q_30_ ≥ 89.19% for B, and a GC content of 43.36% for Y and 44.62% for B. A total of 56,969 unigenes were assembled from *Pfu.* mantle data, among which 21,610 unigenes were annotated using public databases (Additional file 1). Differential expression analysis identified 385 up-regulated unigenes and 227 down-regulated unigenes in Y compared with B with thresholds of |log_2_ fold change (FC) | ≥2 and false discovery rate (FDR) < 0.001 (Table 1, Additional file 2, Fig. 3a).

**Table 1:** Statistics for Illumina transcriptomic sequencing of *Pfu.* mantle edge tissues

Feature	Number	Ratio
Total Number of Unigenes	56,969	–
N50 Length of Unigenes	1004	–
All Annotated	21,610	Annotated ratio 37.9%
Clean Reads num. of T01	35,164,932	Mapped Reads Ratio 80.96%
Clean Reads num. of T02	28,132,519	Mapped Reads Ratio 77.09%
All DEGs (T02/T01) (| log_2_ (fold change) | ≥ 2 and false discovery rate < 0.001)	up-regulated 385 down-regulated 227	–

T01 = mantle edges of black shell pearl oyster (B); T02 = mantle edges of yellow shell pearl oyster (Y)

**Fig. 3:**
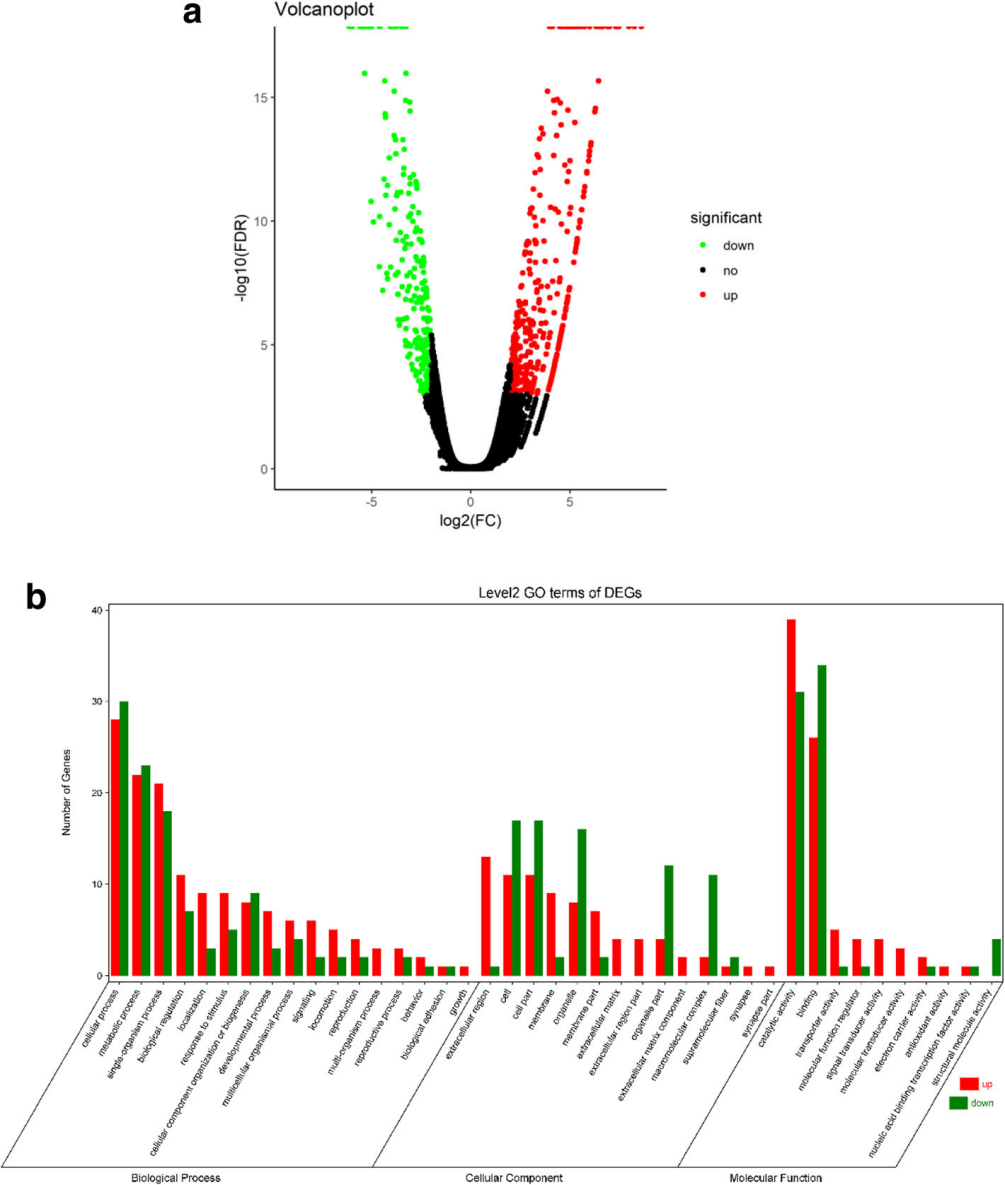
**a** Volcano plot of differentially expressed genes (DEGs) in mantle edge tissues from black and yellow shell pearl oysters (threshold = | log_2_ (fold change) | ≥2 and FDR < 0.001). **b** GO enrichment analysis at level 2 for DEGs for three main categories (Biological Process, Cellular Component and Molecular Function). Green represents down-regulation and red represents up-regulation in Y compared with B.

Among all 21,610 annotated unigenes from pearl oyster mantle edge tissue, the most highly expressed genes are cytochrome c oxidase and glycine-rich protein (GRP). GRPs (c78345.graph_c0, c52407.graph_c0, and c78344.graph_c0) showed higher expressions in B, while the GRP (c52407.graph_c0) was identified as *shematrin-2*. These highly expressed GRPs showed no significant differences in transcriptome data.

Genes encoding shell matrix proteins, nacre proteins, mantle proteins, calmodulins, and some oxidases exhibited significant differences (|log_2_FC| ≥2 and FDR < 0.001) (Table 2). KEGG pathway analysis indicated that differentially expressed genes (DEGs) enriched in phototransduction (ko04744, *p* = 0.018310), melanogenesis (ko04916, *p* = 0.043589), calcium signalling pathway (ko04020, *p* = 0.065512) and tyrosine metabolism (ko00350, *p* = 0.437642) may be correlated with yellow shell pigmentation. All DEGs were also subjected to GO functional analysis, and the three main categories at level 2 of DEGs were illustrated in Fig. 3b. GO terms (Additional file 4) including rhodopsin mediated signalling pathway (GO:0016056), deactivation of rhodopsin mediated signalling (GO:0016059), metarhodopsin inactivation (GO:0016060), adaptation of rhodopsin mediated signalling (GO:0016062) and phototransduction (GO:0007602) might be related to retinal/rhodopsin metabolism. Some DEGs also were significantly enriched (*p* < 0.05) in GO terms including serine-type endopeptidase inhibitor activity (GO:0004867), endopeptidase inhibitor activity (GO:0004866), and peptidase inhibitor activity (GO:0030414; Fig. 4). Additionally, scavenger receptor cysteine-rich domain superfamily protein (SRCR) and rhodopsin (c47803.graph_c0, c67987.graph_c0) were significantly up-regulated in Y transcriptome (Additional file 2).

**Table 2:** Selected DEGs in *Pfu.* edge mantle tissues

GeneID	Nr/ Swissprot annotation	FDR	log_2_FC	Significant or not	qPCR validation
c50039.graph_c0	nacre protein [*Pinctada fucata*]	2.22E-16	6.446008706	up, yes	down
c62280.graph_c0	nacre protein (N16.3) [*Pinctada fucata*]	2.08E-13	3.357226519	up, yes	up
c70362.graph_c0	nacre protein [*Pinctada fucata*]	6.97E-06	2.154265848	up, yes	up
c56018.graph_c0	RecName: Full = Uncharacterized shell protein 12 [*Pinctada margaritifera*]	1.95E-05	2.221337962	up, yes	up
c61883.graph_c0	RecName: Full = Uncharacterized shell protein 1; [*Pinctada margaritifera*]	4.61E-07	2.475769919	up, yes	up
c68187.graph_c0	RecName: Full = Shell matrix protein, partial [*Mytilus californianus*]	0	4.11289221	up, yes	down
c45445.graph_c0	mantle gene 6 [*Pinctada fucata*]	3.08E-05	−3.177947326	down, yes	up
c55873.graph_c0	mantle protein 12 [*Pinctada fucata*]	1.05E-07	−2.845558652	down, yes	up
c59543.graph_c0	mantle gene 1 [*Pinctada fucata*]	0	−3.904743757	down, yes	up
c77975.graph_c1	mantle gene 4 [*Pinctada fucata*]	0	−4.001364161	down, yes	down
c50108.graph_c0	calmodulin, partial [*Paracyclopina nana*]	9.34E-05	2.025942795	up, yes	up
c66278.graph_c0	Calmodulin [*Crassostrea gigas*]	1.98E-05	−2.130307651	down, yes	down
c72378.graph_c0	putative calmodulin [*Schistosoma mansoni*]	0.996557005	0.302184851	up, no	up
c64073.graph_c0	Astacin [*Crassostrea gigas*]	0	4.97505774	up, yes	up
c73606.graph_c0	Putative tyrosinase-like protein tyr-3 [*Crassostrea gigas*]	0	5.148959225	down, yes	up
c76659.graph_c1	Sulfide: quinone oxidoreductase, mitochondrial [*Crassostrea gigas*]	4.63E-11	−2.626911255	down, yes	down
c66049.graph_c0	cellular retinoic acid/retinol binding protein [*Metapenaeus ensis*]	1.07E-05	−2.382034648	down, yes	down
c75964.graph_c0	K07424 cytochrome P450, family 3, subfamily A [EC:1.14.14.1]	0.005094036	−1.489849812	down, no	down

c72378.graph_c0 and c75964.graph_c0 in Table 2 are not significant DEGs

**Fig. 4:**
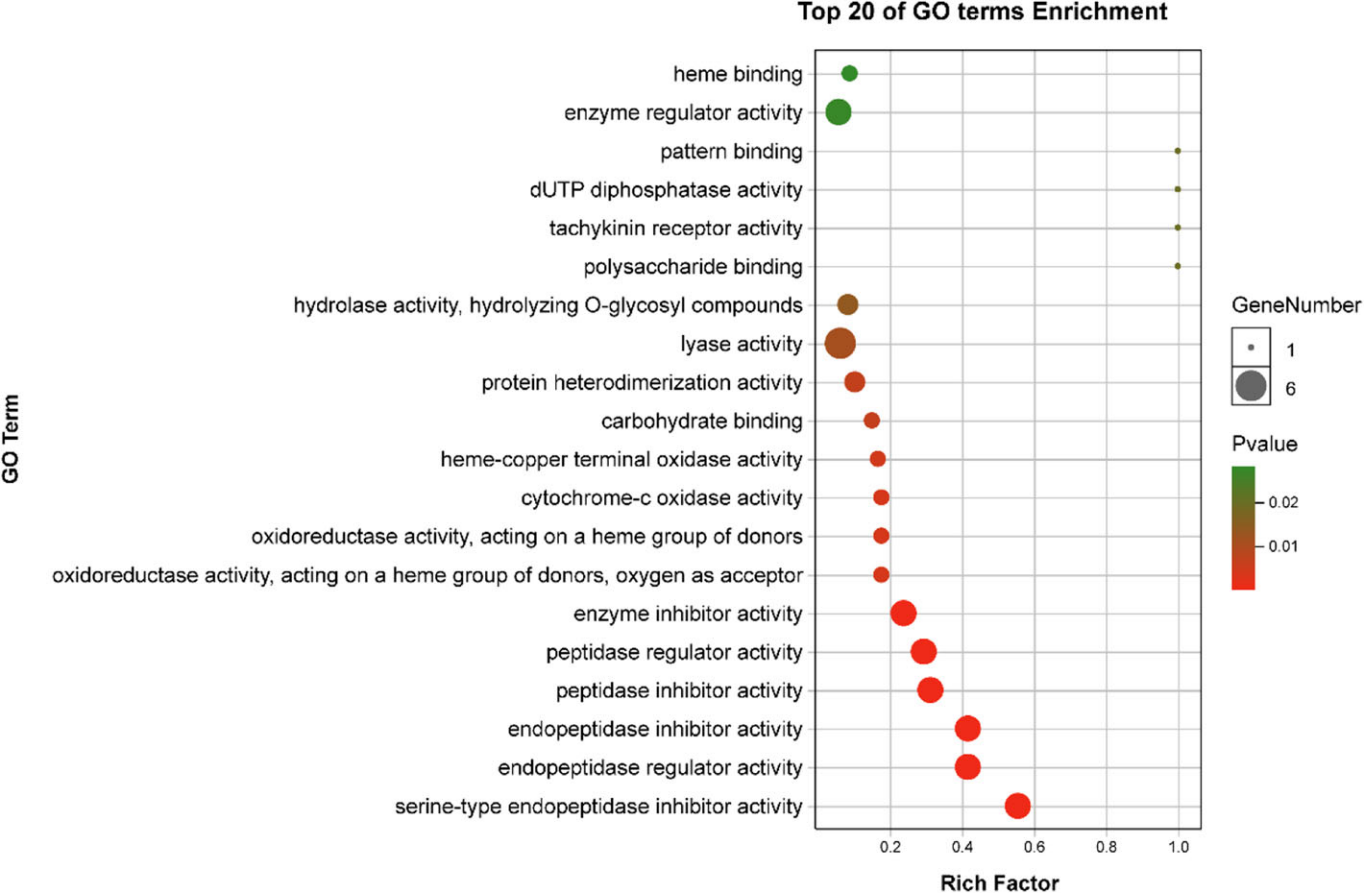
Top 20 enriched GO terms related to DEGs in the Molecular Function category

### DEPs identified by label-free proteome analysis

**Fig. 5:**
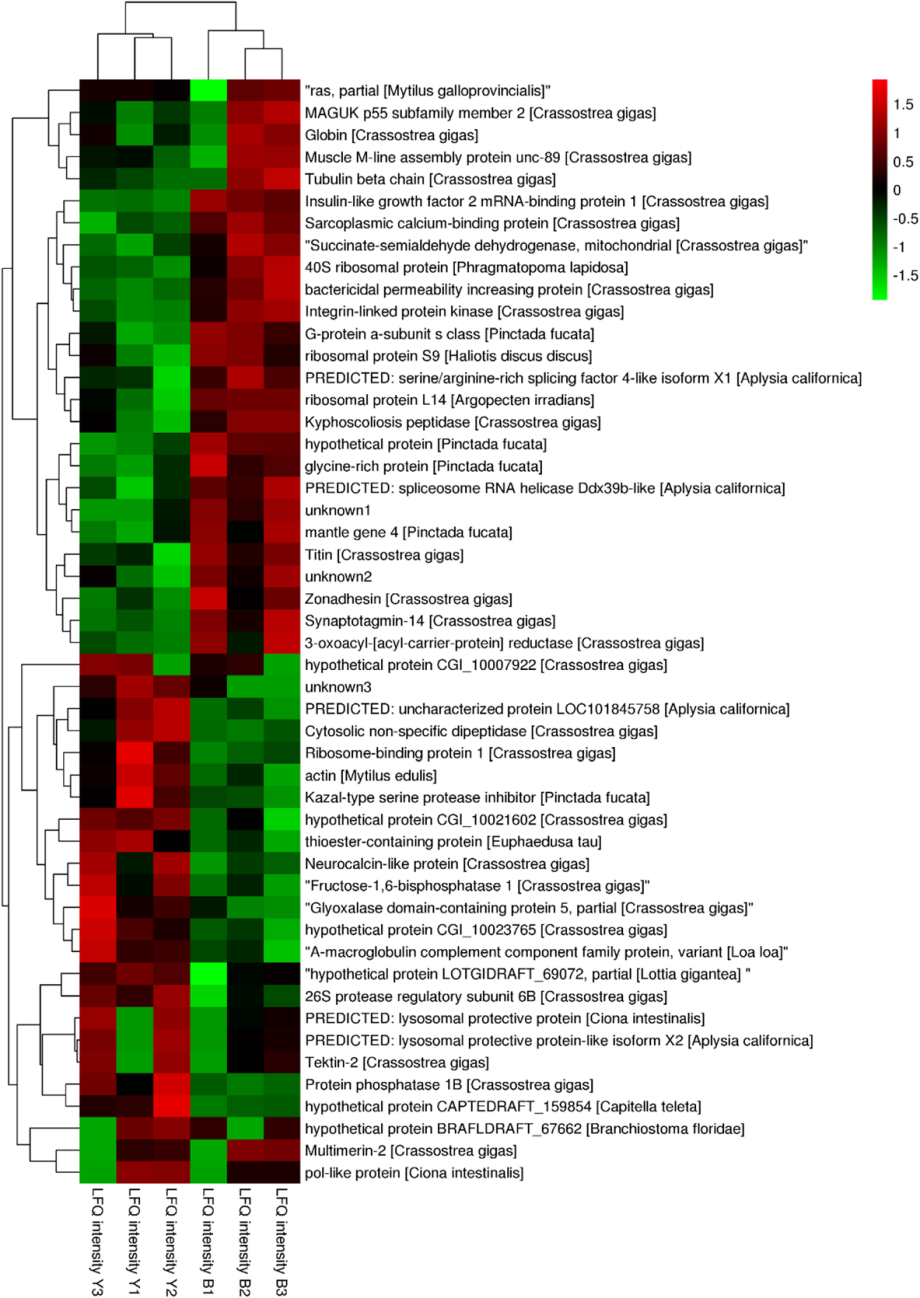
Heatmap of significant DEPs based on label-free proteome data. FC (Y/B) ≥1.2 and *p* < 0.05 served as criteria for significant differences. Green represents lower expression, red represents higher expression, and “unknown” refers to unigenes without annotation.

Following MaxQuant analysis, peptide sequences were searched against the transcriptome reference dataset, yielding 1769 proteins (Additional file 3), including 24 significant up-regulated proteins and 27 significant down-regulated proteins in Y compared with B (Table 3). Of these, 1684 proteins were quantified and another 85 proteins were qualitatively analysed using a label-free proteomics approach. The criteria for DEPs were FC ≥1.2 and t-test *p*-value < 0.05. A heatmap of DEPs was generated (Fig. 5, Additional file 3) that shows significantly higher expression of mantle gene 4 (c77975.graph_c1) and GRP (c52407.graph_c0) in group B samples, and significantly higher expression of a Kazal-type serine protease inhibitor (SERP) (c51835.graph_c0), actin protein and a thioester-containing protein (TEP) (c54125.graph_c0) in Y.

**Table 3:** Statistics for *Pfu.* mantle edges proteome data

Feature	Number
Identified proteins	1769
Quantified proteins	1684
Qualitated proteins	85
Quantified proteins	1684
DEPs (FC > 1.2)	938
DEPs (FC > 1.2 and *p* < 0.05)	51

### qPCR validation of genes related to biomineralisation, calcium regulation, and retinol metabolism

**Fig. 6:**
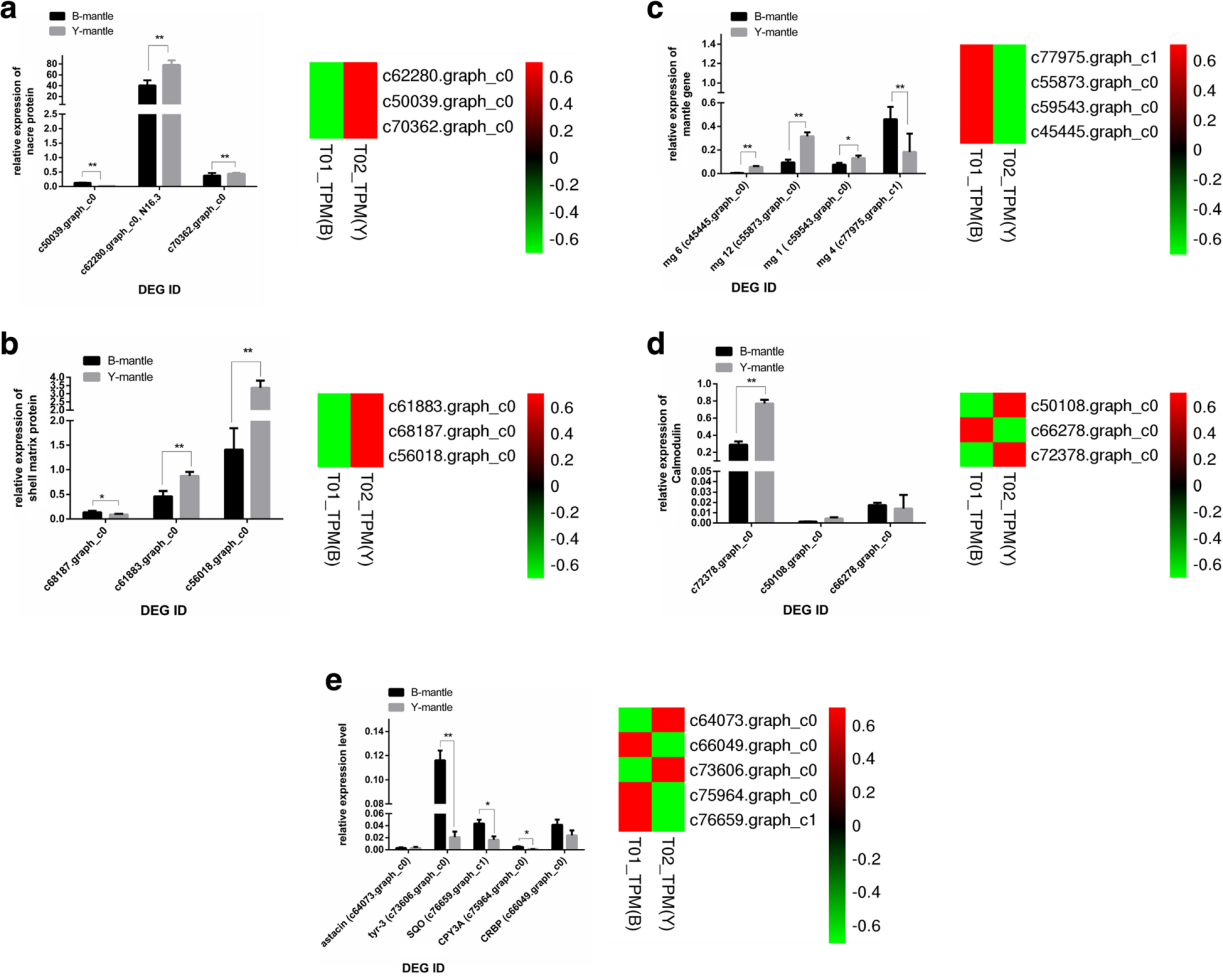
**a** qRT-PCR validation of transcriptome data for nacre proteins. **b** qRT-PCR validation of transcriptome data for shell matrix proteins. **c** qRT-PCR validation of transcriptome data for mantle genes. **d** qRT-PCR validation of transcriptome data for calmodulin genes. **e** qRT-PCR validation of transcriptome data for oxidase genes

Expression of DEGs related to shell colour and biomineralisation was validated by qRT-PCR, as shown in Fig. 6. Three nacre proteins showed significant differential expression in the transcriptomes, and two yielded consistent qRT-PCR validation results (Fig. 6a). Label-free proteome analysis also revealed higher expression of nacre protein N16.3 in Y. Two out of three shell proteins showed similar transcriptome and qRT-PCR results (Fig. 6b), as did one of the four mantle genes (Fig. 6c), among which *mantle gene 4* displayed lower expression in Y according to all three quantification methods. Two calmodulin proteins (c72378.graph_c0 and c50108.graph_c0) showed identical trends in transcriptome, proteome and qRT-PCR validation methods. CYP3A, which was not detected in proteome analysis, was down-regulated in Y according to transcriptome and qRT-PCR results. *Tyr-3* showed higher expression in B according to qRT-PCR, but lower expression in B according to transcriptome sequencing.

In summary, 12 of 18 (66.7%) selected DEGs showed concordance in transcriptome and qRT-PCR methods. Although only five of 18 selected DEGs were detected in the label-free proteome analysis, four of them (80%) showed concordance in transcriptome, qRT-PCR and proteome analyses. Nacre protein N16.3 and calmodulins (c72378.graph_c0, c50108.graph_c0) were highly expressed in Y according to transcriptomic, qRT-PCR, and label-free proteomic quantification, while GRP *shematrin-2* (c52407.graph_c0), mantle gene 4 and sulphide: quinone oxidoreductase (c76659.graph_c1) were highly expressed in B.

### Analysis of correlations between transcriptome and label-free proteome data

**Fig. 7:**
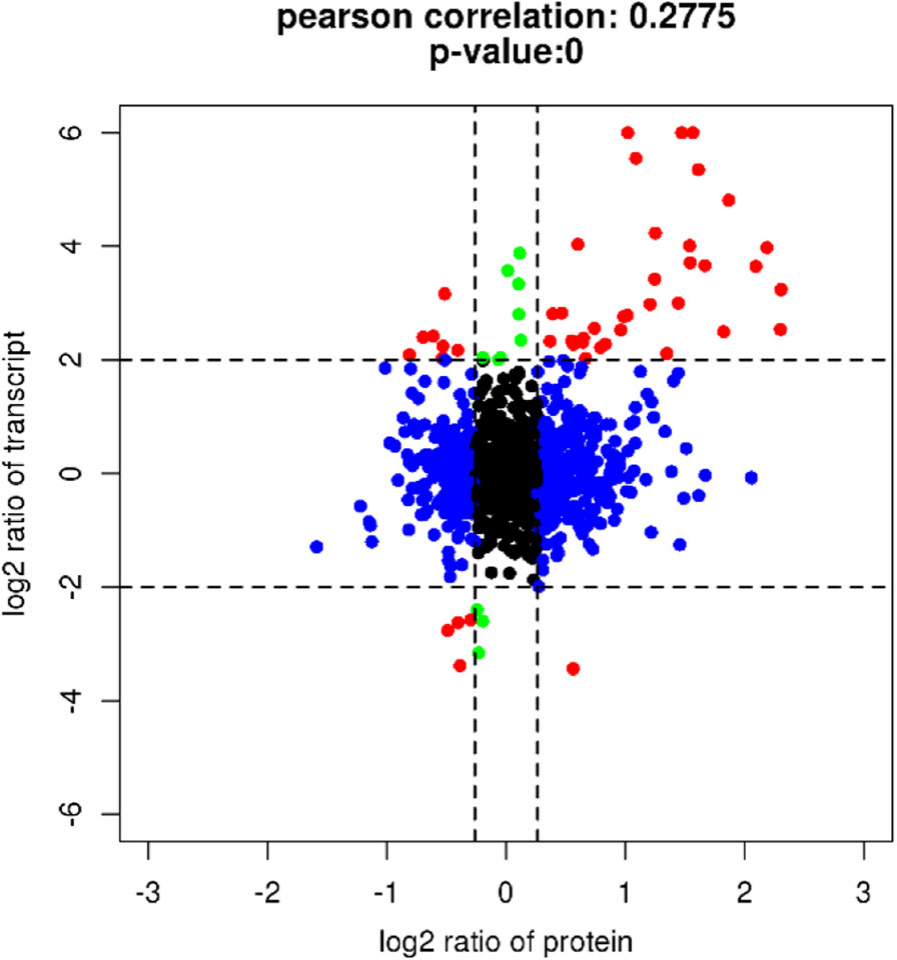
Correlation of transcriptome and proteome differences between Y and B. The first row represents parts 1,2 and 3. The second row represents parts 4,5 and 6. The third row represents parts 7, 8 and 9

Global analysis of the association between transcriptome and proteome data was performed, and the Pearson correlation coefficient for the two omics approaches was 0.2775 (Additional file 5), as shown in Fig. 7. The bulk of unigenes were divided in the central part (part 5).

In parts 1, 4 and 7, 217 of 1008 unigenes were down-regulated at the protein level regardness of mRNA level. Four of seven unigenes distributed in part 1 were presumed to be extracellular structural proteins. GRP *shematrin-2* (c52407.graph_c0), sulphide: quinone oxidoreductase (c76659.graph_c1) and F-type lectin (c65133.graph_c0) in part 7 showed lower expression in Y at two omics levels.

Analysis of parts 3, 6 and 9 revealed that 346 of 1008 unigenes were up-regulated at the protein level. A total of 30 unigenes in part 3 showed higher expression levels in Y at both mRNA and protein levels, including EF-hand domain protein-like calmodulin and peptidase family protein. These proteins are mostly involved in amino acid transport and metabolism, posttranslational modification, signal transduction mechanisms according to KOG annotation, and some have unknown functions. Three calmodulins (c50108.graph_c0, part 3; c75759.graph_c1, part 6; c72314.graph_c0, part 6) involved in GO terms metarhodopsin inactivation and adaptation of rhodopsin mediated signalling were also up-regulated in the Y proteome. In part 6, a homeobox protein transcript factor that inhibits beta-carotene 15, 15′-monooxygenase (BCMO1) activity in human intestine [32] and Kazal-type serine protease inhibitor (c51835.graph_c0) were elevated in the Y proteome. Enzymes such as L-amino-acid oxidase (EC:1.4.3.2; part 5) and maleylacetoacetate isomerase (EC:5.2.1.2; part 6) in tyrosine metabolism was also higher in the Y proteome.

Besides, other mollusc shell structural proteins including N66 matrix protein (c61538.graph_c0), sushi domain (c76538.graph_c0), von Willebrand factor domain (c72946.graph_c0), EGF domain (c68361.graph_c0), chitin-binding domains (c66442.graph_c0) [14], and actin, tubulin, and myosin heavy chain were identified in the proteome. In part 2, N66 matrix protein (c61538.graph_c0), a key organic matrix component facilitating prismatic and nacreous layer formation in the silver-lipped pearl oyster *Pinctada maxima* [33], displayed higher expression in Y at two omics levels.

## Discussion

### The yellow shell trait in *Pfu.* Might be influenced by both carotenoids and melanin

Shell colour differences in Y and B may be due to the combined effects of carotene and melanin, based on higher TCC values and lower tyrosinase-like protein 3 in the mantle of Y. TCC has been linked to shell colour [27] and immunity [28] in noble scallop *Chlamys nobilis*. Meanwhile, Zheng et al. [27] found that scallops with orange shell and orange muscle were significantly correlated with higher TCC values (*p* < 0.05) compared with brown shell organisms. TCC values in different tissues from different mollusc species (μg/g) have been reported previously. TCC in viscera (110 μg/g) of *Haliotis discus* was higher than that in gonad (62 μg/g) and adductor muscle (2 μg/g), and TCC in viscera (67 μg/g) of *Modiolus modiolus difficans* was higher than that in gonad (19 μg/g) and adductor muscle (1 μg/g) [34]. TCC in gonad was higher than mantle, adductor muscle and gill in noble scallop, ranging from 0.73 to 59.85 μg/g [27]. Absorption of beta-carotenoids by animals such as molluscs depends on the food chain and carotenoids are believed to be essential for reproduction in marine animals [35]. Thus, high accumulation of carotenoids in the digestive gland and gonads of pearl oysters is consistent with the results of previous studies. Karnaukhov [36] found carotenoids in neurons of the gastropod mollusc *Lymnaea stagnalis*, and higher TCC values in foot tissue of Y in this study may be related to the pedal ganglion.

Conversion of dietary β-carotene into biologically active products such as retinal and rhodopsin is quite complex. Scavenger receptor proteins family have been proved to function in uptake of carotenoids [32] and Liu et al. [37] has found that down-regulation of scavenger receptor class B-like-3 can decrease blood carotenoid in scallop. Whether the up-regulation of SRCR in Y transcriptome was related to accumulation of caronoid is uncertain. Retinoids was the first carotenoid metabolic transformation in many animal tissues [32, 35]. Herein, some DEGs were enriched in retinal metabolism-related pathways including phototransduction, and rhodopsin-related terms such as metarhodopsin inactivation, according to KEGG and GO enrichment analysis. This may imply different carotenoid-related signalling pathways in B and Y.

Although tyr-3 was up-regulated in Y according to transcriptome data, it was found to be down-regulation in Y by qRT-PCR. Tyrosine metabolism is related to melanogenesis (ko 04916), and tyrosine is a differential metabolite between Y and B (data not shown). High expression of *tyr-3*, a key enzyme for melanogenesis and colour formation [38], might contribute to accumulation of black pigment in B.

The identified signalling pathways/terms related to carotenoid metabolites and the melanogenesis pathway, together with the TCC results, indicated that yellow shell colour might be due to the combined effects of carotene accumulation and melanin shortage in Y. Further research involving the identification of carotenoids in *Pfu.* is clearly needed.

### Genes involved in calcium regulation might affect melanogenesis in the mantle edge of *Pfu*

Calcium regulation might be related to melanogenesis (ko04916). Enrichment analysis of significant DEGs illustrated three calmodulin unigenes enriched in melanogenesis, and qPCR validation results followed the same trend as those of transcriptome analysis. Two of them were also identified in label-free proteome analysis with higher expression in Y. Buffey et al. found that the calcium and calmodulin signalling system has an inhibitory influence on melanogenesis in murine B16 melanoma cells [39], and calcium has an inhibitory effect on pre-tyrosinase activity [40].

Paradoxically, we found that CaMK II and calpain were up-regulated in Y at the mRNA level but down-regulated at the protein level. Kenji et al. [41] found that calpain plays a positive regulatory role in melanogenesis in mouse B16 melanoma cells. Thus, we predict that lower expression of calpain protein in Y might down-regulate melanogenesis, but the exact function of CaMK II in melanogenesis remains elusive. Together, higher Calmodulin and lower calpain might influence melanogenesis in the mantle edge of *Pfu*.

### Biomineralisation and calcium regulation may contribute to yellow shell colour pigmentation

We predict that yellow pigmentation might be connected to special shell structure and biomineralisation-related genes. Lemer et al. [4] suggested that biomineralisation is related to pigmentation in *Pinctada margaritifera*, and some of the identified DEGs also showed differential expression in the proteome.

Many genes related to biomineralisation, such as GRPs, matrix proteins, calmodulins, sushi domains, von Willebrand domains, EGF domains, chitin-binding domains, collagen alpha chain, c-type lectin, ferritin, and others [8, 14] were detected in label-free proteome analysis, demonstrating the differential expression profiles of these genes in black- and yellow-shelled oysters. Interestingly, GRPs were highly expressed in the group B transcriptome and proteome, but only significantly at the proteome level. This could indicate the accumulation of shell proteins or matrix proteins. GRP *shematrin-2*, functioning in mineralisation in *Pfu.* [2].

GRP and mantle gene 4 were significantly up-regulated in the proteome of group B, while Kazal-type SERP was higher in Y. Wang et al. [42] found that mantle gene 4 in *Pfu.* can increase mineral deposition. The Kazal-type SERP results was consistent with GO enrichment of DEGs in the main Molecular Function category (Fig. 4), implying different serine-type endopepetide activity in Y and B. SERP was highly expressed in albino phenotype black-lipped pearl oyster *Pinctada margaritifera* compared with the normal black phenotype [4]. The high level of Kazal-type SERP in yellow oysters might be related to protecting the organic matrix in the shell against exogenous digestive enzymes [14].

In summary, yellow-shell pearl oysters possess higher TCC than wild black-shell oysters. Furthermore, genes related to phototransduction, rhodopsin metabolism, melanogenesis, calcium signalling and biomineralization were differentially expressed in Y and B organisms according to transcriptomic, label-free proteomic, and qRT-PCR analyses. Thus, the yellow shell colour phenotype in *Pfu.* might be due to contributions from three processes: carotenoid metabolite-related signalling pathways, calcium regulation, and biomineralisation.

## Conclusions

A total of 21,610 annotated unigenes were obtained in transcriptomic and 1769 proteins were identified in proteomic. The yellow phenotype of pearl oysters, which possess higher total carotenoids, may be related to phototransduction, retinal and rhodopsin metabolism, tyrosine metabolism, calcium metabolism, melanogenesis and biomineralisation. These results provide insights into exploring the relationships between calcium metabolism, biominesalization and yellow shell colour pigmentation.

## Methods

### Preparation of tissues from pearl oysters

F2 families of *Pfu.* artificially bred for yellow shell colour by our lab, were established in 2014 and cultured at the Marine Biology Research Station in Daya Bay, Chinese Academy of Sciences (Shenzhen, China). Black shell pearl oysters (B) and yellow shell pearl oysters (Y) aged 1.5 years were randomly sampled from one F2 family (Fig. 1). Tissue samples including digestive gland, gonad, gill, foot, heart, adductor muscle and mantle edge were collected from each oyster individual. Part of each sample was stored with RNA/DNA sample protector (TaKaRa, Japan) for RNA abstraction, another portion was frozen in liquid nitrogen for protein abstraction, and the rest was lyophilised using a vacuum freeze-dryer (ALPHA 1–4 LD plus, Martin Christ, Germany) for TCC determination. Animals used in this study were not endangered and were treated in accordance with regulations.

### Total carotenoid extraction and TCC determination

The same tissues from seven yellow shell and seven black shell oysters were mixed and dried in a freeze-dryer (Martin Christ, Germany) for 20–24 h. The different tissue mixtures were ground with pestles and mortars that sterilised at 180 °C in an oven dryer before (Fuma, China). Total carotenoids were extracted according to Yanar’s and Zheng’s method [27, 43]. Each tissue sample in triplicate was homogenized and dissolved in 1.2 ml acetone, and incubated with shaking at 200 rpm/min for 2 h in a dark room at 25 °C. After centrifuging the tissue extracts at 2400 g for 5 min, the supernatant (1 ml) was scanned in an ultraviolet-visible spectrophotometer (TU-1810, Persee, China). Total carotenoid content (TCC) was calculated using an extinction coefficient E _(1%,1cm)_ of 1900 at an absorption wavelength of 480 nm [27, 43] by the following equation:

$$ \mathrm{TCC}\ \left(\upmu \mathrm{g}/\mathrm{g}\ \mathrm{dry}\ \mathrm{weight}\right)={\mathrm{A}}_{480}.\mathrm{y}\ {.10}^4/\left({{\mathrm{E}}_{1\mathrm{cm}}}^{1\%}.\mathrm{W}\right). $$

where y is the volume of supernatant (ml) and W is the dry weight of tissue powder (g).

Student’s t-tests were applied to compare the mean values of each tissue; *p* < 0.05 was considered significant (*), and *p* < 0.01 was considered highly significant (**).

### Generation of a reference dataset from *Pfu.* Mantle transcriptome data for proteomic analysis

#### cDNA libraries construction and Illumina sequencing using hi-Seq 4000

Total RNA was abstracted using a Mollusc RNA kit (Omega, USA) according to the manufacturer’s protocol, and then checked with a 1% agarose gel. RNA purity, concentration and integrity were investigated using a NanoPhotometer spectrophotometer (IMPLEN, CA, USA), a Qubit 2.0 Fluorimeter (Life Technologies, CA, USA), and an Agilent Bioanalyzer 2100 system (Agilent Technologies, CA, USA), respectively.

Total RNA (3 μg per sample, 1 μg per individual) from mantle edges of three yellow shell and black shell pearl oysters was used to construct two libraries using an NEB Next Ultra RNA Library Prep Kit for Illumina (NEB, USA) following the manufacturer’s recommendations. Firstly, mRNA was enriched from total RNA with polyT oligo-attached magnetic beads, then broken randomly into fragments with fragmentation buffer. Secondly, first-strand cDNA was then synthesised using random hexamer primer and M-MLV reverse transcriptase. Second-strand cDNA was then obtained using DNA Polymerase I and RNase H. Double-stranded cDNA was purified using AMPure XP beads (Beckman Coulter, Beverly, USA) to select fragments of 150–200 bp. Thirdly, Illumina paired-end adaptors were then ligated to DNA fragments after adenylation of 3’ends, and fragments were incubated with USER Enzyme (NEB) for 15 min at 37 °C, followed by 5 min at 95 °C. PCR was then performed with Phusion High-Fidelity DNA polymerase, Universal PCR primers and Index (X) Primer. Finally, PCR products were purified and library quality was assessed using an Agilent Bioanalyzer 2100 system. The two cDNA libraries were sequenced using an Illumina Hi-Seq 4000 platform.

#### De novo assembly and functional annotation

Clean reads were obtained by removing low-quality reads and reads containing adapter and/or poly -N from raw data. Meanwhile, Q_20_, Q_30_, GC content and sequence duplication level of clean reads were calculated. De novo assembly of *Pfu.* mantle transcripts was conducted with Trinity software [44] with min_kmer_cov set to 2 by default and all other parameters set as default values. Clean reads for each sample were mapped back to the assembled transcript reference and transcript quantification was performed with RSEM (http://deweylab.biostat.wisc.edu/rsem/). Transcripts Per Million (TPM) values based on read counts and transcript lengths were used to evaluate the expression level of each unigene. Transcripts were functionally annotated against Nr database [45], Universal Protein knowledgebase (UniProt) [46], Gene Ontology (GO) [47], Clusters of Orthologous Groups of proteins (COG) [48], euKaryotic Ortholog Groups (KOG) [49], eggNOG4.5 [50], and Kyoto Encyclopedia of Genes and Genomes database (KEGG) databases [51] using BLAST [52] (E-value ≤1e^− 5^). KEGG Orthology (KO) analysis of unigenes was performed using the KOBAS 2.0 web server (http://kobas.cbi.pku.edu.cn/ [53]). Prediction of coding sequences (CDS) and peptide amino acid sequences of unigene peptides was conducted using TransDecoder software (https://github.com/TransDecoder). After searching for profiles contained in the Pfam database [54] the presence of conserved protein domains was assessed with HMMER 3.0 (http://www.hmmer.org/ [55]). Peptide sequence datasets were then used for protein identification.

#### Functional enrichment analysis of differentially expressed genes (DEGs)

Expression level of unigenes were evaluated by TPM values based on read counts and transcript lengths. Differential expression analysis was conducted using the EBSeq R package with thresholds of |log_2_FC| ≥2 and FDR < 0.001. The resulting FDR values were adjusted based on the posterior probability of being differentially expressed (PPDE). GO and KEGG enrichment analyses were performed with the topGO R package (http://www.bioconductor.org/packages/2.11/bioc/html/topGO.html) [56] and KOBAS 2.0 respectively with a hypergeometric test.

### Liquid chromatography-mass spectrometry (LC-MS) analysis of the mantle proteome

#### Protein abstraction and concentration determination

Nine oysters from each group (Y and B) were used for label-free proteome analysis. Mantle edge tissues from three individuals were mixed as one sample, and labelled B1–3 and Y1–3 for each group. These six samples were homogenised with an MP Fastprep-24 Automated Homogenizer (MP Biomedicals, USA) in SDT buffer (pH 7.6) comprising 4% sodium dodecyl sulphate (SDS), 1 mM dithiothreitol (DTT) and 100 mM TRIS-HCl. Homogenates were sonicated, boiled for 15 min, then centrifuged at 14,000 g for 40 min at 4 °C. Supernatants were filtered with 0.22 μm filters (Millipore), and filtrates were quantified with a BCA Protein Assay Kit (Bio-Rad, USA). SDS-polyacrylamide gel electrophoresis (SDS-PAGE) was performed for each sample (20 μg) on a 12.5% gel (constant current 14 mA, 90 min). Protein bands were visualised by staining with Coomassie Brilliant Blue R^− 250^.

#### Preparation of mantle peptides

Filter-aided sample preparation (FASP Digestion) was performed as described previously [57]. Briefly, 200 μg of protein from each sample was mixed with 30 μl SDT buffer, and the reducing agent DTT and other compounds were removed using UA buffer (8 M Urea, 150 mM TRIS-HCl, pH 8.0) by repeated ultrafiltration with a 10 kDa ultrafiltration tube (Sartorius, Germany). Samples were incubated for 30 min in darkness to block reduced cysteine residues using 100 μl iodoacetamide (IAA). After washing with 100 μl UA buffer three times and 100 μl 25 mM NH_4_HCO_3_ buffer twice, the proteins were digested with 4 μg trypsin (Promega, USA) in 40 μl 25 mM NH_4_HCO_3_ buffer overnight at 37 °C. Finally, the resulting peptides were collected as a filtrate, desalted on C18 cartridges (standard density, bed internal diameter = 7 mm, volume = 3 ml; Sigma), concentrated with vacuum centrifugation (Eppendorf Concentrator Plus, Germany) and reconstituted in 40 μl 0.1% (v/v) formic acid.

#### High-performance liquid chromatography (HPLC)

LC analysis of each peptide mixture was performed on an EASY-nLC System (Thermo Fisher Scientific) in buffer A (0.1% formic acid) with 10 cm capillary columns (internal diameter = 75 μm) containing 3 μm resin (Thermo Fisher Scientific). Peptides were eluted with a linear gradient from 0 to 55% buffer B over 110 min, 55–100% buffer B for 5 min, and 100% buffer B for 5 min at a flow rate of 300 nl/min controlled by IntelliFlow technology.

#### LC-MS and data analysis

LC-MS analysis of eluates was performed on a Q Exactive mass spectrometer (Proxeon Biosystems, now Thermo Fisher Scientific) coupled to an Easy nLC System for 60 min. The mass spectrometer was operated in positive ion mode. MS data were acquired using a data-dependent top 10 mode with dynamic selection of the 10 most intense peaks of each survey scan (300–1800 m/z) for high collision dissociation (HCD) fragmentation. Survey scans were acquired at a resolution of 70,000 at m/z 200, and the resolution of HCD spectra was set to 17,500 at m/z 200, with an isolation width of 2 m/z. The automatic gain control (AGC) target was set to 3*e^6^, and the maximum inject time was 10 ms. The dynamic exclusion duration was 40.0 s, the normalised collision energy was 30 eV, and the underfill ratio was defined as 0.1%. The instrument was run with peptide recognition mode enabled.

MS data were analysed using MaxQuant software version 1.5.3.17 [58]. Proteome-wide quantification with label-free approaches was performed according to the Max-label-free quantification (LFQ) intensity determination and normalisation procedure (Max-LFQ) [59]. Briefly, MaxLFQ algorithms are included within the MaxQuant software suite and MaxLFQ is a generic method for label-free quantification with standard statistical tests of quantification accuracy for each of thousands of quantified proteins (FDR < 0.01). Protein quantification was performed according to the ‘unique plus razor peptides’ mode and intensity determination was calculated using the full peak volume. All raw files were searched against the CDS sequences of the *Pfu.* mantle edges transcriptome. The statistical significance of differences between yellow shell and black shell oysters was analysed by t-tests via LFQ values for three samples in each group, and values with FC ≥1.2 and *p* < 0.05 were considered significant.

### Validation of DEGs by quantitative real-time PCR (qRT-PCR)

A total of 18 DEGs annotated as nacre proteins, shell matrix proteins, mantle proteins, calmodulins, and oxidases were selected for qPCR validation. Mantle edge tissues from five yellow shell and black shell pearl oysters were prepared for total RNA abstraction after homogenisation using a T18 instrument (IKA, Germany). Total RNA was isolated using a Mollusc RNA kit (Omega) as described above then treated with ReverTra Ace qPCR RT master mix with gDNA Remover kit (Toyobo, Japan) to prepare cDNA template. qRT-PCR analysis was performed on a Roche LightCycler 480 Real-time PCR System (Roche, Switzerland) using SYBR Green Real-time PCR Mix (QPK-201, Toyobo). Reactions (10 μl) contained 0.4 μl forward primer, 0.4 μl reverse primer, 5 μl SYBR mix, 3.2 μl ddH_2_O, and 1 μl cDNA (diluted 1:5 in water). All samples were analysed with four replicates in a 384-well plate. PCR amplification was conducted with 40 cycles at 95 °C for 10 s, 55 °C for 15 s, and 72 °C for 15 s, in single acquisition mode. Crossing point (Cp) or cycler threshold (Ct) was monitored and recorded when the reaction fluorescence first rose above the background level. Relative expression of target genes was calculated using the 2^-ΔCt^ transformation [60]. A *p-*value < 0.05 (*) indicated a significant difference and *p* < 0.01 (**) indicated a highly significant difference. Primers for amplification of target genes and the 18S rRNA internal reference gene are listed in Table 4.

**Table 4:** Primers used for qRT-PCR validation of *Pfu.* mantle edge tissues transcriptome data

Primer name	Gene name	Sequences (5′-3′)
18S-F	18S	CGTTTCAACAAGACGCCAGTAG
18S-R		ACGAAAAAAAGGTTTGAGAGACG
c50039-RT-F	Nacre protein	TTCATCGCTACCATTACAACGG
c50039-RT-R		TCGTCGGAAGATTACAGCATTC
c62280-RT-F	Nacre protein, N16.3	TCTGTAGATACGCTTGGTCTCC
c62280-RT-R		TTCCGTTACCGTTGTCATCATC
c70362-RT-F	Nacre protein	CTTGGACAGGCACAGACTCA
c70362-RT-R		CTGAATGGTGTTCTTCGGCAAT
c68187-RT-F	Shell matrix protein	CCAGTGCTTCCAATACCAAGG
c68187-RT-R		TCCAGTGCGGTTGTATGTGTA
c61883-RT-F	Shell protein 1	ATTCAGTTCCGTACCTGTTCCT
c61883-RT-R		ACATTGTTGGCGTCTAGTCTCT
c56018-RT-F	Shell protein 12	TAGGAGGAGCACAGCACTTG
c56018-RT-R		ACGAGGTTGACTGTAGGTGTTA
c55873-RT-F	Mantle gene 12	CGTTCATAGACTGCCAACATG
c55873-RT-R		TAAGACTGACGCCGACTGT
c77975-RT-F	Mantle gene 4	TCCGAGAGGTAATGTTGTCTAC
c77975-RT-R		CCGTTGTACTCAGTGGAAGAA
c45445-RT-F	Mantle gene 6	CCTTTACGCATTGGTTGATACG
c45445-RT-R		TGAAGAGTTGGCTGAAGTGTTC
c59543-RT-F	Mantle gene 1	AAGGATGGTGGAGAAGGACAA
c59543-RT-R		TCGTAAGAAGCTAGTTCACAGG
c72378-RT-F	Calmodulin (CaM)	ATTGGGACAGAACCCAACAG
c72378-RT-R		GCTTCCCGGATTTCTTCTTC
c50108-RT-F	Calmodulin (CaM)	CGAGAAGCTTTCCGAGTGTT
c50108-RT-R		TCCGTCCCCATCTAAATCAG
c66278-RT-F	Calmodulin (CaM)	GAGACCGTCGGCATCAGTAT
c66278-RT-R		TGAAGAAGTATCAGCGGAGGAT
c64073-RT-F	astacin	ATCACCGCCATCTGTTCCT
c64073-RT-R		TCCGCTTCTACTACTTCCTGTA
c73606-RT-F	tyr-3	TGGTCAGGTCTGTAGGTTGTG
c73606-RT-R		TGGATGTTCTTCGGTGATGGT
c51674-RT-R		TCCTCGGATCTTGTCTGACTTC
c76659-RT-F	Sulfur quinone oxidoreductase	CGACTACGACGCTAATCCTCT
c76659-RT-R		ATGATGTTGCCTTCACTCTTCC
c75964-RT-F	CYP3A	GCACGGTCCAACCTTAATACTG
c75964-RT-R		AGGCGACAAGAGATTCAAGAAC
c66049-RT-F	CRBP	GATGAAGTCACGGCAGATGG
c66049-RT-R		CTAGTGATGACCGATTCAGGAT

tyr-3, tyrosinase-like protein 3; CYP3A, cytochrome P450 3A; CRBP, cellular retinol binding protein

### Analysis of correlations between transcriptome and proteome sequencing data

Log_2_ transformation of FC data from transcriptome and label-free proteome was applied to analyse correlations between the two omics approaches, and the results were visualised using scatter plots generated by R (| log_2_FC | ≥2 for mRNA, and FC ≥1.2 for proteins) [61]. Genes were divided into nine parts according to log_2_ transformation of FC at mRNA and protein levels.

## Additional files

Additional file 1: 21,610 annotated unigenes in transcriptome. A total of 21,610 unigenes can be annotated with public databases. (XLS 9388 kb)

Additional file 2: All differential unigenes compared T02 (Y) with T01 (B). There are 10,557 differential unigenes including 612 significant differentially expressed genes (DEGs). (XLS 5782 kb)

Additional file 3: All differential proteins compared Y with B (quantitative). There are 1684 differential proteins quantified including 51 significant differentially expressed genes (DEPs), and another 85 proteins were qualitatively analysed. (XLS 1602 kb)

Additional file 4: GO enrichment analysis at level 2 for DEGs for three main categories (Biological Process, Cellular Component and Molecular Function) (XLS 235 kb)

Additional file 5: Unigenes divided into nine parts for analysis of correlations between transcriptomic and label-free proteomic. (XLS 562 kb)

## Data Availability

Transcriptome data has been uploaded to the NCBI SRA database (SRR8357272 for B, SRR8357273 for Y); Label-free proteome data has been uploaded as ProteomeXchange Datasets and data are available from ProteomeXchange under identifier PXD012211.

## Abbreviations

BCMO1: Beta-carotene 15, 15′-monooxygenase
CaM: Calmodulin
CaMK II: Calcium/calmodulin-dependent protein kinase II
COG: Clusters of Orthologous Groups of proteins
CRBP: Cellular retinol binding protein
CYP3A: Cytochrome P450 3A
DEGs: Differentially expressed genes
DEPs: Differentially expressed proteins
DOPA: 3,4-dihydroxyphenylalanine
FC: Fold change
FDR: False discovery rate
GRP: Glycine-rich protein
KEGG: Kyoto Encyclopedia of Genes and Genomes
KOG: EuKaryotic Ortholog Groups
LFQ: Label-free quantification
SERP: Kazal-type serine protease inhibitor
SQO: Sulphide: quinone oxidoreductase
SRCR: Scavenger receptor cysteine-rich domain
TAC: Total antioxidant capacity
TCC: Total carotenoid content
TEP: Thioester-containing protein
TPM: Transcripts per million reads
tyr-3: Tyrosinase-like protein 3
